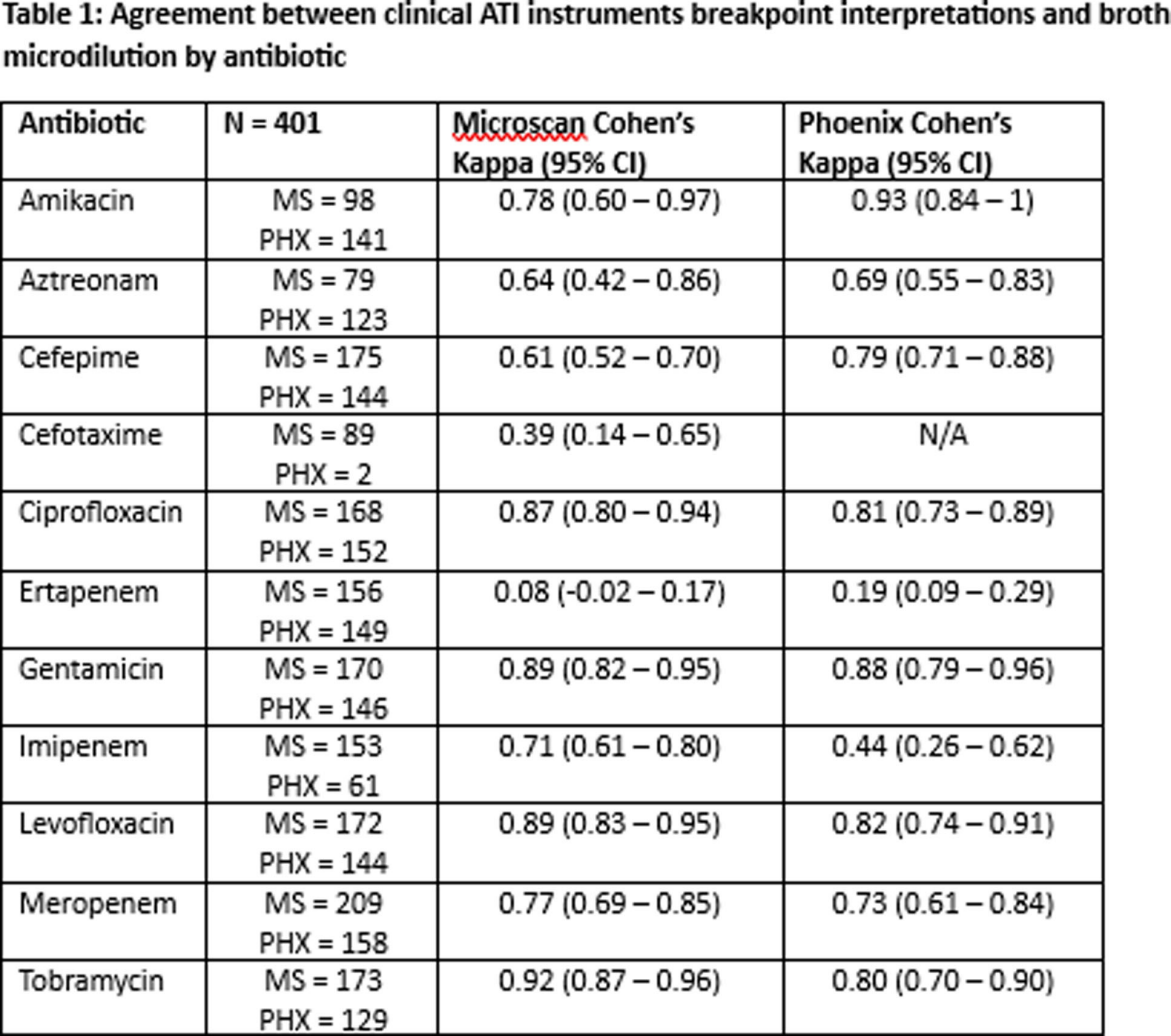# 187 Vaccine effectiveness of Bivalent RSVpreF against laboratory-confirmed RSV stratified by age and comorbidities in nursing home resident

**DOI:** 10.1017/ash.2026.10579

**Published:** 2026-06-23

**Authors:** Erin Christenson, Sarah Petersen, Daniel Muleta, Samantha Mathieson

**Affiliations:** 1 Tennessee Department of Health; 2 TN Department of Health

## Abstract

**Background** Use of automated antimicrobial susceptibility testing (AST) instruments are common in clinical microbiology laboratories, in part due to the quick turnaround time and automated result reading. Given the wide variability in validation and each individual instrument’s algorithms and parameters, AST data between clinical laboratories is not guaranteed to be comparable. Accurate AST is critical to ensure appropriate antimicrobial prescribing practices. In Tennessee little is known about the comparability of AST testing amongst clinical labs. This study aims to quantify differences in clinical automated testing instrument (ATI) performance when compared to broth microdilution (BMD). Methods Clinical laboratory AST data from Phoenix (PHX) and Microscan (MS) instruments were obtained for carbapenem-resistant Enterobacterales (CRE) and carbapenem-resistant Acinetobacter baumannii (CRAB) via the Multi-site Gram-Negative Surveillance Initiative. Data were received from the residents of seven select counties in Tennessee from 2019–2024. Identified isolates were sent to the Tennessee State Public Health Laboratory’s Antimicrobial Resistance Lab Network (ARLN) program for additional testing, including BMD AST, which was used as the reference standard. Agreement between ATIs and BMD breakpoint interpretations were measured using a weighted Cohen’s Kappa calculated in SAS 9.4. Results There were 401 isolates included in this study. Eleven antibiotics were evaluated for agreement between MS and BMD, and ten were evaluated for agreement between PHX and BMD, shown in Table 1. Six antibiotics and four antibiotics showed lower agreement to BMD for PHX and MS respectively. Both MS and PHX showed near perfect agreement (κ<0.81) for four antibiotics. Additionally, MS and PHX both showed moderate agreement (κ<0.41) for five antibiotics, two of which were imipenem and meropenem. Agreement between ATI and BMD was poor for ertapenem across both MS and PHX, with the data showing clinical ATI are over-reporting resistance compared to BMD. Agreement between MS and BMD was also poor for cefotaxime (0.39). Conclusion While this study only evaluated a subset of isolates tested at the clinical laboratory, it still demonstrates that clinical AST instruments results are not necessarily comparable, with half of the antibiotics evaluated showing moderate to poor agreement when compared to BMD. The poor agreement reported for ertapenem is particularly concerning, as these represent overreported carbapenem resistance, which impacts treatment options, transmission-based precaution implementation, and unnecessary confirmatory testing through ARLN. This study demonstrates the need for laboratories utilizing commercial AST instruments to perform frequent quality control checks to ensure instruments are functioning within an acceptable performance standard range.

**Figure:**